# Circulating Blood Monocyte Subclasses and Lipid-Laden Adipose Tissue Macrophages in Human Obesity

**DOI:** 10.1371/journal.pone.0159350

**Published:** 2016-07-21

**Authors:** Tal Pecht, Yulia Haim, Nava Bashan, Hagit Shapiro, Ilana Harman-Boehm, Boris Kirshtein, Karine Clément, Iris Shai, Assaf Rudich

**Affiliations:** 1 Department of Clinical Biochemistry and Pharmacology, Faculty of Health Sciences, Ben-Gurion University of the Negev, Beer-Sheva, Israel; 2 The National Institute of Biotechnology in the Negev (NIBN), Ben-Gurion University of the Negev, Beer-Sheva, Israel; 3 Diabetes Unit, Soroka University Medical Center and Ben-Gurion University of the Negev, Beer-Sheva, Israel; 4 Department of Surgery A, Soroka University Medical Center and Ben-Gurion University of the Negev, Beer-Sheva, Israel; 5 Institute of Cardiometabolism and Nutrition, ICAN, Assistance Publique-Hôpitaux de Paris, Pitié-Salpêtrière hospital, Nutrition department, F-75013, Paris, France; 6 Department of Public Health, Faculty of Health Sciences, Ben-Gurion University of the Negev, Beer-Sheva, Israel; University of Cambridge, UNITED KINGDOM

## Abstract

**Background:**

Visceral adipose tissue foam cells are increased in human obesity, and were implicated in adipose dysfunction and increased cardio-metabolic risk. In the circulation, non-classical monocytes (NCM) are elevated in obesity and associate with atherosclerosis and type 2 diabetes. We hypothesized that circulating NCM correlate and/or are functionally linked to visceral adipose tissue foam cells in obesity, potentially providing an approach to estimate visceral adipose tissue status in the non-surgical obese patient.

**Methods:**

We preformed *ex-vivo* functional studies utilizing sorted monocyte subclasses from healthy donors. Moreover, we assessed circulating blood monocyte subclasses and visceral fat adipose tissue macrophage (ATM) lipid content by flow-cytometry in paired blood and omental-fat samples collected from patients (n = 65) undergoing elective abdominal surgery.

**Results:**

*Ex-vivo*, NCM and NCM-derived macrophages exhibited lower lipid accumulation capacity compared to classical or intermediate monocytes/-derived macrophages. Moreover, of the three subclasses, NCM exhibited the lowest migration towards adipose tissue conditioned-media. In a cohort of n = 65, increased %NCM associated with higher BMI (r = 0.250,p<0.05) and ATM lipid content (r = 0.303,p<0.05). Among patients with BMI≥25Kg/m^2^, linear regression models adjusted for age, sex or BMI revealed that NCM independently associate with ATM lipid content, particularly in men.

**Conclusions:**

Collectively, although circulating blood NCM are unlikely direct functional precursor cells for adipose tissue foam cells, their increased percentage in the circulation may clinically reflect higher lipid content in visceral ATMs.

## Introduction

With the expansion of adipose tissue in obesity, adipose tissue macrophages (ATM) were found to accumulate and support tissue remodeling by storing lipids and scavenging dead adipocyte remains, giving rise to a unique lipid-laden ATM subpopulation–adipose tissue foam cells [1–3]. In human obese patients, particularly in the omental visceral fat depot [4], ATM lipid content increases by several folds compared to lean patients. Moreover, clinically, the number of adipose tissue foam cells in omental fat associates with patients’ indicators of insulin resistance and systemic inflammation [3]. Furthermore, functionally, adipose tissue foam cells isolated from high fat–fed obese mice induce insulin resistance when incubated with adipose tissue explants from lean mice [3]. Jointly, these data suggest that the presence of lipid-laden ATMs is a feature of obese visceral adipose tissue that contributes to obesity-associated metabolic morbidity. Yet, the cellular source of adipose tissue foam cells, and clinically—how to identify high visceral adipose tissue foam cells in the non-surgical obese patient, remain unclear.

Despite emerging evidence for ATM proliferative capacity [5], circulating blood monocytes are largely considered a predominant source for ATM accumulation in chronic obesity [6]. Interestingly, human blood monocytes constitute a heterogeneous cell population that can be sub-divided according to the surface expression pattern of CD14 (LPS co-receptor), and CD16 (low-affinity FcγIII receptor): CD14^+^CD16^-^ (classical monocytes, CM), CD14^+^CD16^+^ (intermediate monocytes, IM), and CD14^dim^CD16^+^ (non-classical monocytes, NCM) [7]. Normally, these three subpopulations account for 75–85%/5-10%/5-10% (CM/IM/NCM, respectively) of the total circulating monocytes [8]. While the CM and IM present more pro-inflammatory capacity, the NCM patrol the blood vessels’ endothelium, and are presumably involved in the innate local tissue surveillance and tissue repair [7, 9, 10]. The murine analogue of CM (7/4^hi^CCR2^+^Ly-6C^hi^CX3CR1^lo^) were shown to be the subclass which is preferentially recruited to sites of tissue inflammation, and are the likely source of classically-activated (M1) macrophages [11, 12]. Yet, a recent study also demonstrates that the NCM murine analogue subclass infiltrates into sights of tissue inflammation [13]. In humans, available studies on the abundance of circulating CM in obesity are inconsistent, reporting either decreased or no change in obese compared to lean persons [14, 15]. On the other hand, the abundance of circulating CD16+ monocyte subpopulations (IM and NCM) was more consistently reported to be increased in obesity and to associate with cardio-metabolic risk factors [4, 15, 16]. Yet, despite the putative close link between monocytes in the circulating blood compartment and ATMs, how this associates with visceral adipose tissue foam cells abundance has not been investigated.

In this study we investigated which of the three monocyte subclasses displays a higher functional capacity to serve as a possible precursor cell for lipid-laden adipose tissue macrophages. For that, we tested human circulating monocyte subclasses’ migration towards adipose tissue conditioned medium, and lipid storage capacity. Moreover, we explored the association between the abundance of circulating blood monocyte subclasses and visceral ATMs lipid content in patients. Since circulating blood NCM have been shown to increase and associate with cardio-metabolic risk parameters in obese patients, we speculated that in obesity they may be more capable than the CM to migrate towards adipose tissue, and that they may exhibit higher capacity to become lipid-laden.

## Methods

### Patients

Samples were collected as part of "The Beer-Sheva cohort ", following procedures detailed elsewhere [3, 17]. All procedures in this study have been conducted in accordance with the guidelines in the Declaration of Helsinki and were approved by Soroka University Medical Center Institutional Review Committee. All patients gave in advance a written informed consent to all procedures. Blood samples were collected in the morning of the operation after an over-night fast. Fat biopsies samples were obtained during elective abdominal surgery (mainly bariatric surgery and elective cholecystectomy), and immediately delivered to the laboratory, where they were processed for further analyses. All biochemical and endocrinological determinations were performed in the central laboratories of the Soroka Academic Medical Center. The exclusion criteria for patients were: i. age>75 years, and ii. the use of anti-inflammatory or thiazolidinedione (TZD) drugs in the past year. Additionally, in this study we employed the "Ben-Gurion University volunteers' cohort" that consists of healthy male volunteers (n = 10, mean age 29.9±4.3 years, BMI 24.5±3.5 kg/m^2^), for blood samples used for *ex-vivo* monocytes studies.

### Isolation of human adipose stromal-vascular cell fraction (SVF)

SVF was isolated from human fat biopsies as we described previously [3]. In brief, human fat biopsies were extracted and minced in 10mL digestion medium containing DMEM with 4.5mM glucose (without phenol red), 10mM HEPES, pH 7.4, and 1% BSA. Collagenase II (C6885, 1 mg/mL; Sigma-Aldrich, St. Louis, Missouri) was added, and minced tissues were incubated at 37°C for 20 minutes in a shaking bath, following removal of large pieces using 250μm mesh. To further separate lipid-laden macrophages from the adipocyte fraction, samples were washed in 10mM EDTA pH 7.4 buffer and centrifuged at 500g for 7 minutes at 4°C to separate floating adipocyte layer from SVF pallets.

### FACS analysis of human ATM

SVF cells were washed, re-pelleted (500g for 5 minutes at 4°C), and re-suspended in 50 uL lysing buffer (Beckman Coulter, Nyon, Switzerland) for 5 minutes on ice. Samples were then washed and suspended in FACS buffer (PBS supplemented with 2%FBS) containing human Fc Block (eBioscience, San Diego, California) and the following conjugated antibodies (10 minutes on ice in the dark): CD45-PE-Cy7, CD64-APC, and CD14-APC-Cy7 (BioLegend, San Diego, California). Thereafter, samples were washed and stained for 20 minutes on ice with BODIPY 493/503 (3μg/mL BODIPY for 5X10^7^ cells, D3922; Invitrogen, Carlsbad, California). Samples were subsequently washed, filtered through 100μm mesh and stained for viability with propidium iodide (0.2μg/mL; Sigma-Aldrich). Stained samples were analyzed by FACS (FACS Canto II; BD Biosciences, San Jose, California). Data was exported as FCS 3.0 files and was further analyzed using the FlowJo software version 10.0.7.

### Adipose tissue RNA extraction and quantitative RT-PCR

Total RNA from frozen human fat tissue was extracted with the RNeasy lipid tissue mini kit (Qiagen, Germantown, MD) and analyzed with Nanodrop. RNA (200ng) was reverse-transcribed with High Capacity cDNA Reverse Transcriptase Kit (Applied Biosystems, Foster City, CA). Taqman system (Applied-Biosystems, Foster City, CA) was used for real-time PCR amplification. Relative gene expression was obtained after normalization by the formula 2^-dCT^, normalized to control genes PPIA and PGK1 using specific primers (**S1 Table**).

### FACS analysis of human blood monocytes

Human blood samples (0.5–3mL) were transferred into 0.1 M EDTA coated 50 mL lab tubes. Leucocytes fraction was obtained after incubating the blood samples for 2.5 minutes in room temperature with BD FACS Lysing Solution (BD bioscience, San Jose, California). Leucocytes were stained with FACS buffer containing the following conjugated antibodies (10 minutes on ice in the dark): CD3-V450, CD19-V450, CD56-V450, HLA-DR1-APC, CD14-APC-Cy7 and CD16-FITC (BD Bioscience). Stained samples were further washed and filtered using 70μm mesh before analyzed by FACS (FACS Canto II; BD Biosciences, San Jose, California) and the FlowJo software.

### Sorting of classical and non-classical human monocyte subpopulations

100 mL blood samples were collected from healthy male volunteers into EDTA blood collections tubes. Peripheral mononuclear cells (PBMC) obtained by gradient centrifugation using Lymphocytes Separation Medium, according to manufactures' instructions (MP Biomedicals, Solon, Ohio), and monocytes were isolated using CD14-microbeads (Miltineyi Biotech, Germany). Monocytes were then stained with FACS buffer containing human Fc Block and the following conjugated antibodies (30 minutes at 4°C in the dark): CD3-VioGreen, HLA-DR1-VioBlue (Miltineyi Biotech, Germany), CD56-BV510 (BioLegend, San Diego, California), CD16-PE and CD14-APC (BD Bioscience). Stained samples were sorted by the FACS ARIA (BD Bioscience) in sterile conditions into polystyrene tubes containing RPMI medium supplemented with 20% FBS, 2% glutamine, 2% antibiotics. Monocytes were selected by gating for relatively small cells with low granularity, which are negative for CD3\CD20 and CD56 (T-, B- and NK-cells markers) following exclusion of CD16+HLADR- cells (which are mostly neutrophils). According to the expression of CD14 and CD16 the classical and non-classical monocyte subpopulations were sorted (CD14^+^CD16^-^ and CD14^dim^CD16^+^, respectively).

### Ex-vivo lipid loading of human monocytes

Sorted monocytes subpopulations were incubated in 96-well uClear, black plate (Greiner Bio One, Kremsmünster, Austria). For monocytes lipid-loading, the subclasses were cultured 24 hours with 200 μM oleic acid or with 0.05% BSA/DMSO vehicle. For monocyte-derived macrophages, the subclasses were cultured for 7 days with human M-CSF (50 ng/ml; PeproTech, Rocky Hill, NJ) with the addition of 10% human visceral conditioned media for the last 2 days. Lipid accumulation was then measured by fixing the cells with 4% formaldehyde followed by staining with BODIPY (1 ug/ml) and DAPI (5 ng/ml; Invitrogen, Carlsbad, California) for 20 minutes in room temperature. Images were acquired in a fully automated and unbiased manner using X40 wide angle lens equipped microscope (Operetta, PerkinElmer, Waltham, Massachusetts). At least 35 fields per well were collected for statistical analysis, which was done using the image analysis software Columbus (PerkinElmer, Waltham, Massachusetts).

### Preparation of adipose tissue conditioned medium

Omental fat tissue biopsy from patients (100mg) were slightly sliced and incubated overnight in Minimum Essential Medium (MEM)-alpha supplemented with 2% Glutamine, 1% antibiotics and 10% FBS (Biological industries, Beit HaEmek, Israel). After 24 hours, fat tissues were washed once, incubated in fresh medium for additional 24 hours, then medium was collected and stored at -80°C until use.

### Monocytes migration assay

Monocytes isolated from 100 mL blood from healthy male donors by CD14 magnetic-microbeads, were stained with conjugated antibodies CD14-APC, CD16-PE (BD Bioscience) and were then loaded to cell culture inserts with a bottom-permeable membrane (Millicell Hanging Cell Culture Inserts 5μm; Millipore, Billerica, Massachusetts), which placed above human visceral adipose tissue conditioned media. For positive control, medium supplemented with 10% FBS was used. After 150 minutes the media from the bottom chamber was collected, migrating cells were collected and analyzed using FACS Canto II.

### Statistical analysis

Patients’ characteristics are presented as median±SD. Given the distribution pattern and/or n of most parameters, we preferred (as detailed in each figure legend) to use the appropriate non-parametric statistical test: Wilcoxon test was used to assess differences between monocyte subclasses from the same donors. Comparison of clinical parameters of %NCM-low versus %NCM-high patients (defined as those with %NCM below or above the median = 13.2%) was performed using Mann-Whitney test, and Spearman test was used for evaluating the correlations of circulating monocytes subclasses percentages with clinical and adipose tissue parameters. All statistical analyses were performed using either SPSS (version 22) or GraphPad software.

## Results

**Fig 1:**
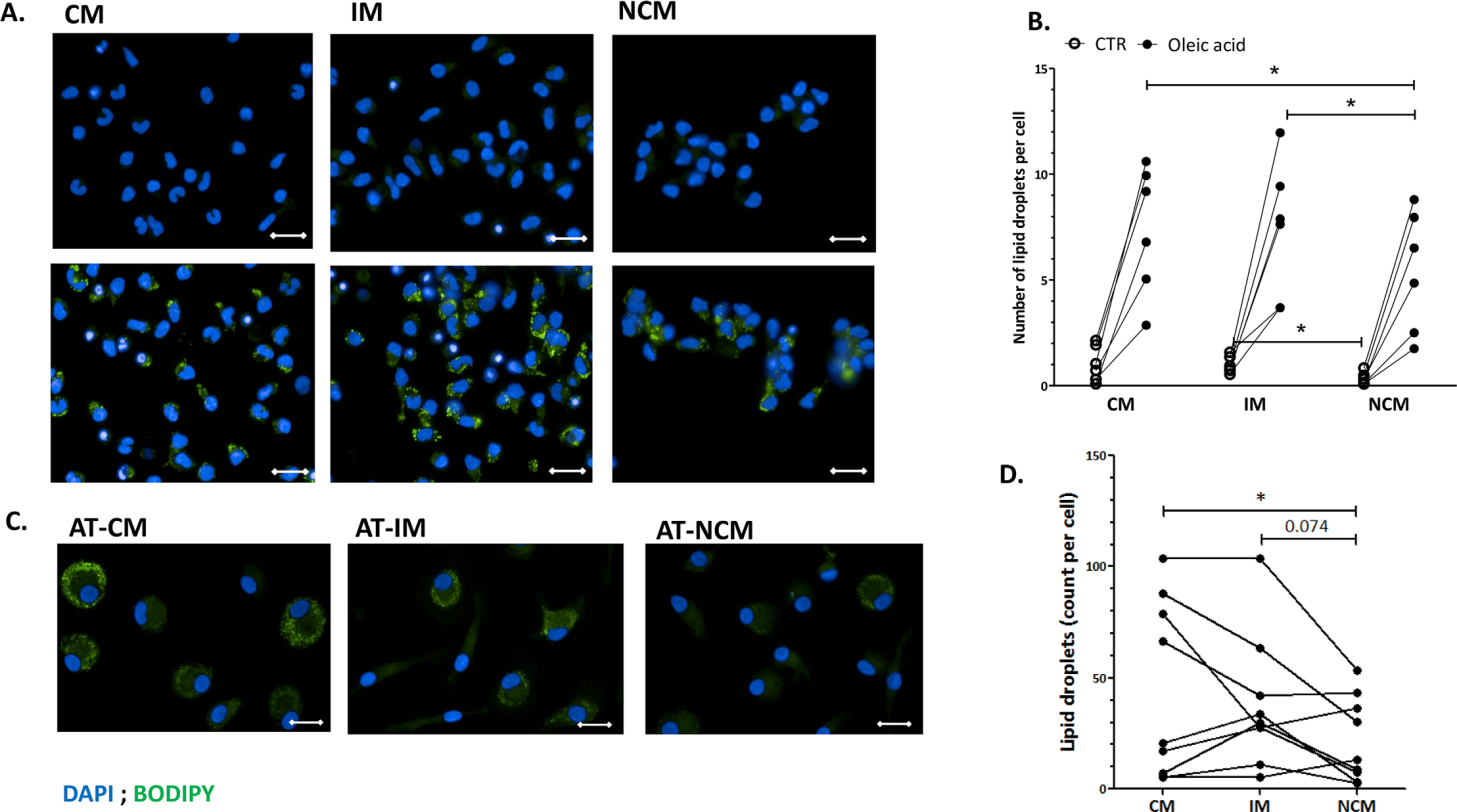
Non-classical monocytes and their derived macrophages exhibit lower lipid accumulation. Monocyte subclasses were sorted from CD14+ enriched PBMC fraction obtained from the blood of healthy male donors. Sorted monocyte subclasses were cultured *ex-vivo* for **A)** 24 hours with oleic acid (200 Μm) or **C)** 7 days with M-CSF (50 ng/ml), supplemented for the last 2 days with 10% human omental adipose tissue conditioned medium, to mimic adipose tissue macrophages. Cells were subsequently fixed and stained with DAPI (blue) and BODIPY (green), to visualize nucleus and lipid droplets, respectively. Representative images for the three subclasses were obtained by Operetta imaging system (**A** and **C**). Bar = 20 μm. Summary of results from 6 or 9 independent donors are shown in **B** and **D**, respectively. Statistical comparison was obtained by the Wilcoxon matched pairs non-parametric test. *p<0.05.

To test the functional potential of NCM as a circulating monocyte subclass that could support lipid-laden ATM accumulation and adipose tissue foam cells formation, we tested *ex-vivo* their ability to: i) accumulate lipids, and ii) migrate towards adipose tissue conditioned media. To that end, the three monocyte subclasses sorted from peripheral blood of healthy male donors were cultured with oleic acid, to mimic a fatty acid -rich environment reminiscent of that within AT. Our results demonstrate that, counter to our hypothesis, NCM exhibited the lowest capacity to accumulate lipids during 24 hours culture without or with 200 μM oleic acid (Fig 1A and 1B and S1 Data). Next, to better mimic omental ATMs and the adipose tissue milieu, sorted monocytes subclasses were first differentiated *ex-vivo* to monocyte-derived macrophages reminiscent of resident ATM (AT-MDMs) by incubating them with human omental conditioned media. As with the monocytes, NCM-derived AT-MDMs accumulated significantly less lipid droplets compared to CM-derived AT-MDMs (p = 0.027), and tended to accumulate less lipids compared to IM AT-MDMs (p = 0.074; Fig 1C and 1D). Jointly, either as monocytes or as macrophages, the NCM subclass did not present a higher lipid storage capacity compared to the other two subclasses.

**Fig 2:**
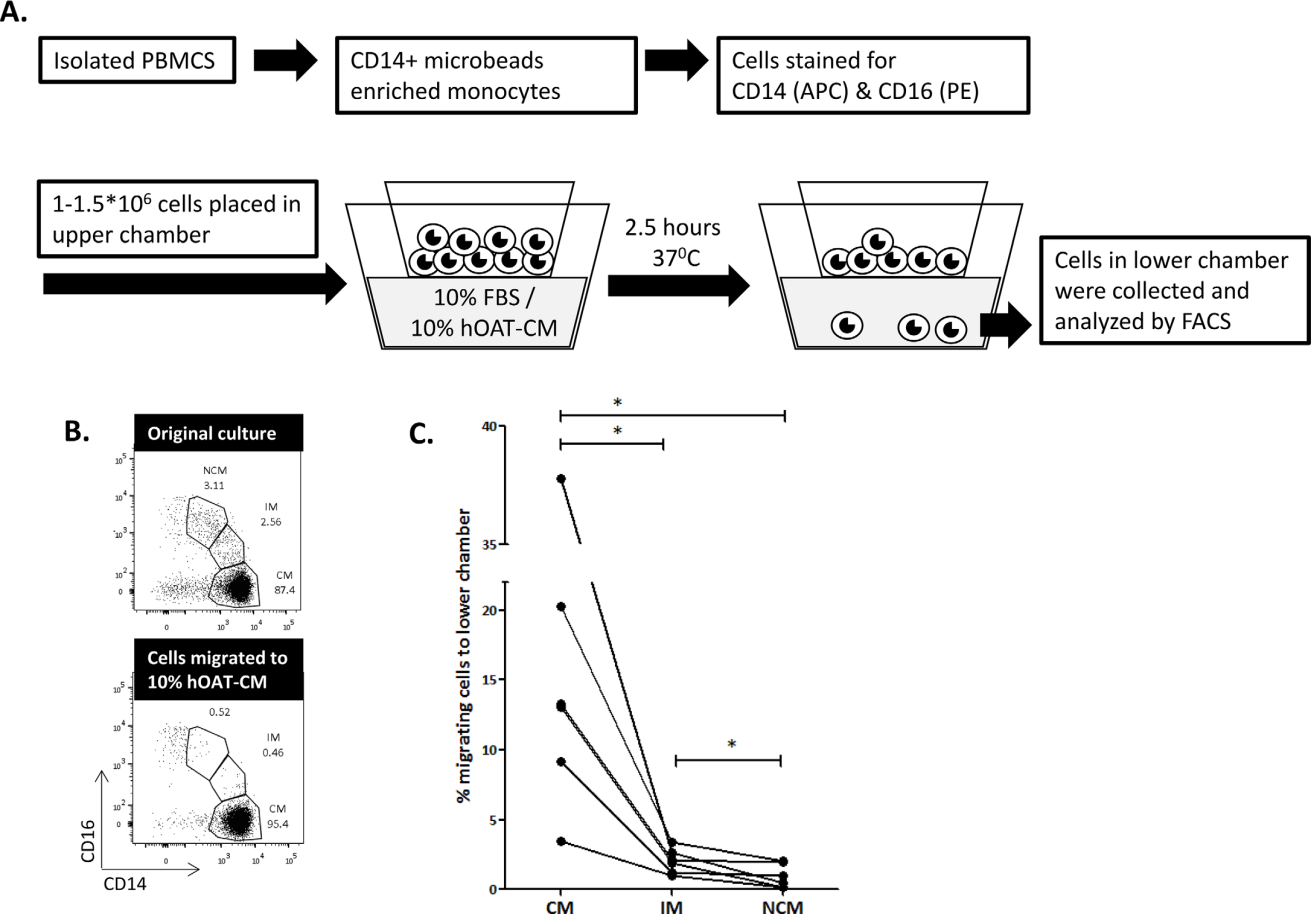
Non-classical and intermediate monocytes migrate less towards conditioned medium of omental adipose tissue. **A)** Following isolation of PBMCs from peripheral blood of healthy male donors, monocytes were enriched by CD14+ magnetic separation and stained for CD14 and CD16. 1–1.5*10^6^ cells were placed in the upper part of a migration chamber, placed on top of RPMI media containing 10% FBS or 10% human omental adipose tissue conditioned media (hOAT-CM). After 2.5 hours in 37°C, cells in the lower chamber were collected, counted, and **B)** analyzed by FACS, and the percentage of migrating cells of each subclass from the original fraction cultured was calculated. Summary of the results obtained from 6 independent donors are shown in **C**. Statistical comparison was obtained by the Wilcoxon matched pairs non-parametric test, *p<0.05.

The three monocyte subclasses were demonstrated to differentially express various chemokine receptors and were reported to have different migration capacities, depending on the environmental signals [18]. To investigate the migratory capacity of the subclasses specifically towards adipose tissue, circulating monocytes were enriched from PBMC fractions by CD14 magnetic microbeads. After staining for CD14 and CD16, cells were incubated for 2.5 hours in a trans-well insert system to test their migration towards 10% human omental adipose tissue conditioned medium (Fig 2A and S1 Data). Of the three monocyte subclasses, NCM presented the lowest migratory capacity towards human omental adipose tissue conditioned-media (calculated as the percentage of monocytes of each subclass from the original fraction cultured, p = 0.031 for both; Fig 2B and 2C). Collectively, the lipid accumulation and migration assay results do not support the notion (and our initial hypothesis) that NCM may functionally constitute a preferred monocyte subclass serving as precursor cells for omental ATMs, or for the specific subclass of lipid-laden adipose tissue foam cells.

**Fig 3:**
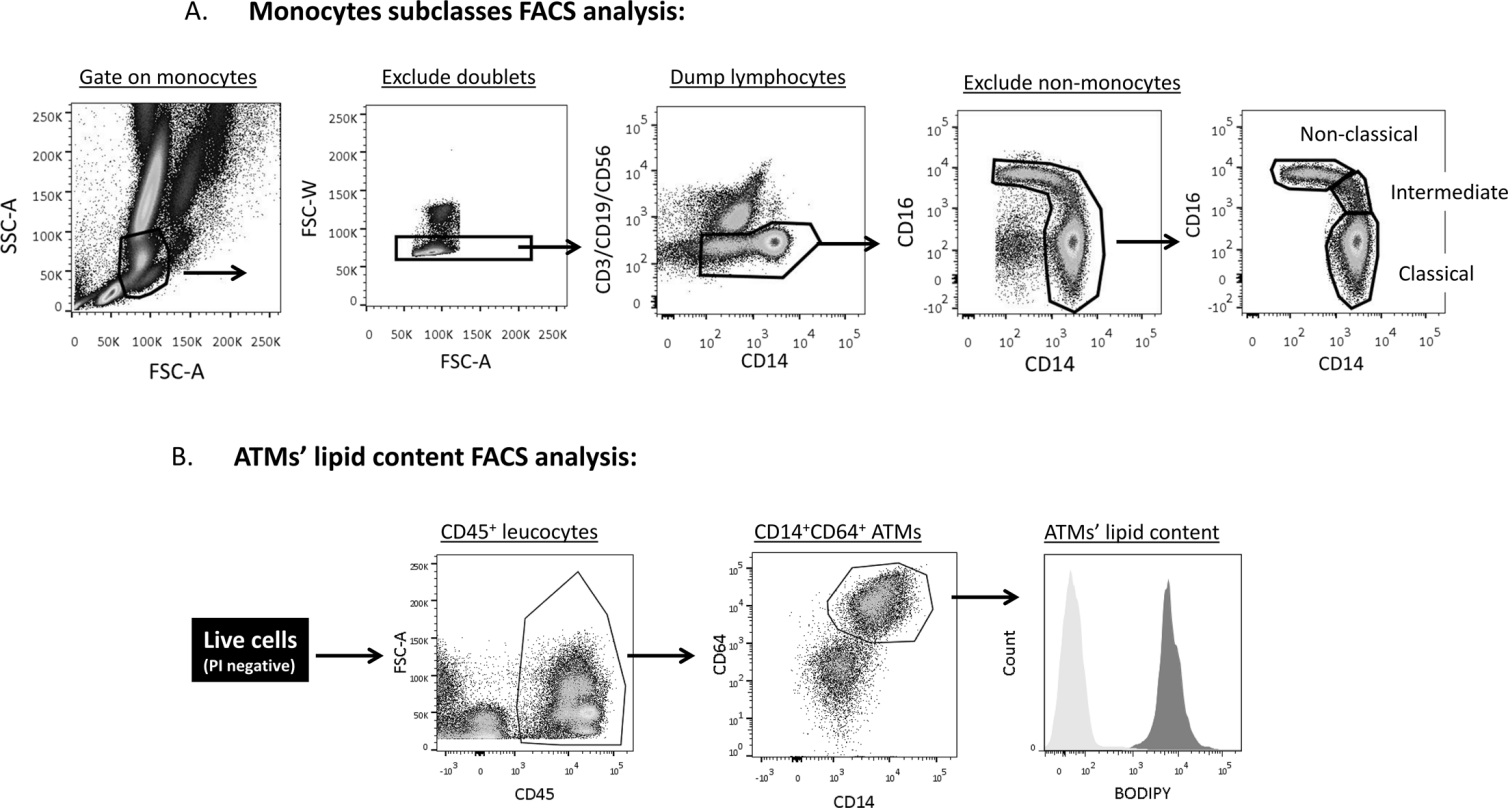
Analyses of circulating blood monocyte subpopulations and adipose tissue macrophage lipid content by FACS. **A)** Circulating blood monocytes subclasses: Following RBC lysis monocytes were identified by gating for small and low granular cells (low side scatter (SSC) and forward scatter (FSC), respectively), and cell doublets were excluded by using Area (FSC-A) vs. Width (FSC-W) signal intensity. T-, B- and NK- lymphocytes were excluded using CD3, CD19 and CD56, respectively. Monocyte subclasses were identified by CD14 and CD16 expression. **B)** Adipose tissue macrophage (ATM) lipid content: SVF was isolated from omental human fat tissues by mechanical and collagenase digestion. Viable ATM were identified as staining negative for propidium iodide (PI), positive for the leucocyte marker CD45, and positive for the macrophage markers CD14 and CD64. ATMs' lipids content was assessed using the neutral lipid fluorescent dye BODIPY readout, gray- unstained sample, black-stained sample.

Since in various studies obesity was associated with increased NCM percentage that associate with cardio-metabolic risk [4, 15, 16], we considered whether NCM abundance in the circulation associated with the abundance of visceral (omental) lipid-laden ATMs in a cohort of patients. For this, paired samples of omental fat and blood were collected from 65 patients undergoing elective abdominal surgeries (median age: 40.0y, 63% females, median BMI: 32.3 Kg/m^2^; Table 1 and S2 Data**)**. The three circulating blood monocyte subclasses were identified in blood samples after red blood cell lysis by gating for small and low granular cells, followed by exclusion of lymphocytes and neutrophils and staining for CD14 and CD16 (Fig 3A). To better define IM we identified a CD16^+^/HLA-DR1^+^ subpopulation (S1 Fig). In parallel, adipose tissue was digested, and ATMs were identified as adipose tissue stromal-vascular cells that were propidium iodide negative, and which stained positively for CD45 (common leucocytes antigen), CD14 and CD64. ATM’s lipid content was determined by the mean fluorescence intensity of the neutral lipid dye BODIPY (Fig 3B), as previously described [3].

**Table 1:** Clinical parameters of Beer Sheva cohort participants (n = 65), stratified by %NCM (low vs. high).

	**All**	**%NCM low (0.9–12.8%)**	**%NCM high (13.2–62.9%)**	**P-value**
**N**	65	32	33	
**NCM**				
% from monocytes	13.2±14.6	5.8±3.2	26.2±13.3	**<0.001**
Count (10^3^/mL)	51.8±121.7	30.6±25.3	111.9±155.2	**<0.001**
**IM**				
% from monocytes	5.0±3.0	3.9±2.9	5.9±2.9	**0.001**
Count (10^3^/mL)	25.3±27.7	19.5±20.2	33.6±33.6	**0.032**
**CM**				
% from monocytes	81.5±16.3	89.2±4.9	66.3±14.9	**<0.001**
Count (10^3^/mL)	385.8±194.5	449.6±209.9	301.4±148.6	**0.005**
**Sex (M/F)**	(24, 41)	(13, 19)	(11, 22)	0.543^#^
**Age (y)**	40.0±15.1	39.0±17.6	42.0±12.4	0.651
**BMI (Kg/m**^**2**^**)**	32.3±7.7	30.9±7.9	33.9±7.5	0.332
**% Obese**	58.5	50.0	66.7	0.173^#^
**Systolic BP (mmHg)**	124.0±16.6	123.5±16.5	124.0±17.0	0.818
**Diastolic BP (mmHg)**	75.0±10.5	79.5±10.4	73.0±10.0	0.061
**Fasting glucose (mg/dL)**	86.0±27.3	86.0±30.4	86.0±23.9	0.987
**HbA1c (%)**	5.5±0.9	5.4±1.0	5.7±0.6	0.157
**Insulin (mIU/L)**	11.5±6.6	12.7±6.5	10.1±6.4	0.130
**HOMA-IR**	2.5±1.7	2.8±1.8	2.2±1.5	0.080
**Diabetes**	4	0	4	**0.042**^#^
**Triglycerides (mg/dL)**	130.0±58.4	135.0±61.9	119.5±53.7	0.171
**HDL (mg/dL)**	41.5±12.3	40.5±14.9	42.5±8.9	0.591
**LDL (mg/dL)**	110.0±27.1	110.0±21.7	110.5±31.6	0.625
**Total cholesterol**	179.0±37.6	182.5±28.9	177.0±46.1	0.789
**SGOT (IU/L)**	24.0±21.2	23.0±19.4	26.5±23.0	0.318
**SGPT (IU/L)**	24.5±43.9	19.5±35.1	28.0±51.8	0.186
**AP (IU/L)**	81.5±32.9	73.0±26.2	86.0±35.1	**0.013**
**CRP (mg/L)**	0.5±2.8	0.4±3.7	0.6±0.7	0.423

Presented median ± S.D., p-value Mann-Whitney test, Abbreviations: %NCM, percentage of NCM from total monocytes; BMI, body mass index; TG, triglyceride; HDL, high density lipoprotein; Tchol, total cholesterol; FPG, fasting plasma glucose; BP, blood pressure; CRP, c-reactive protein.

^#^Chi-square test.

**Fig 4:**
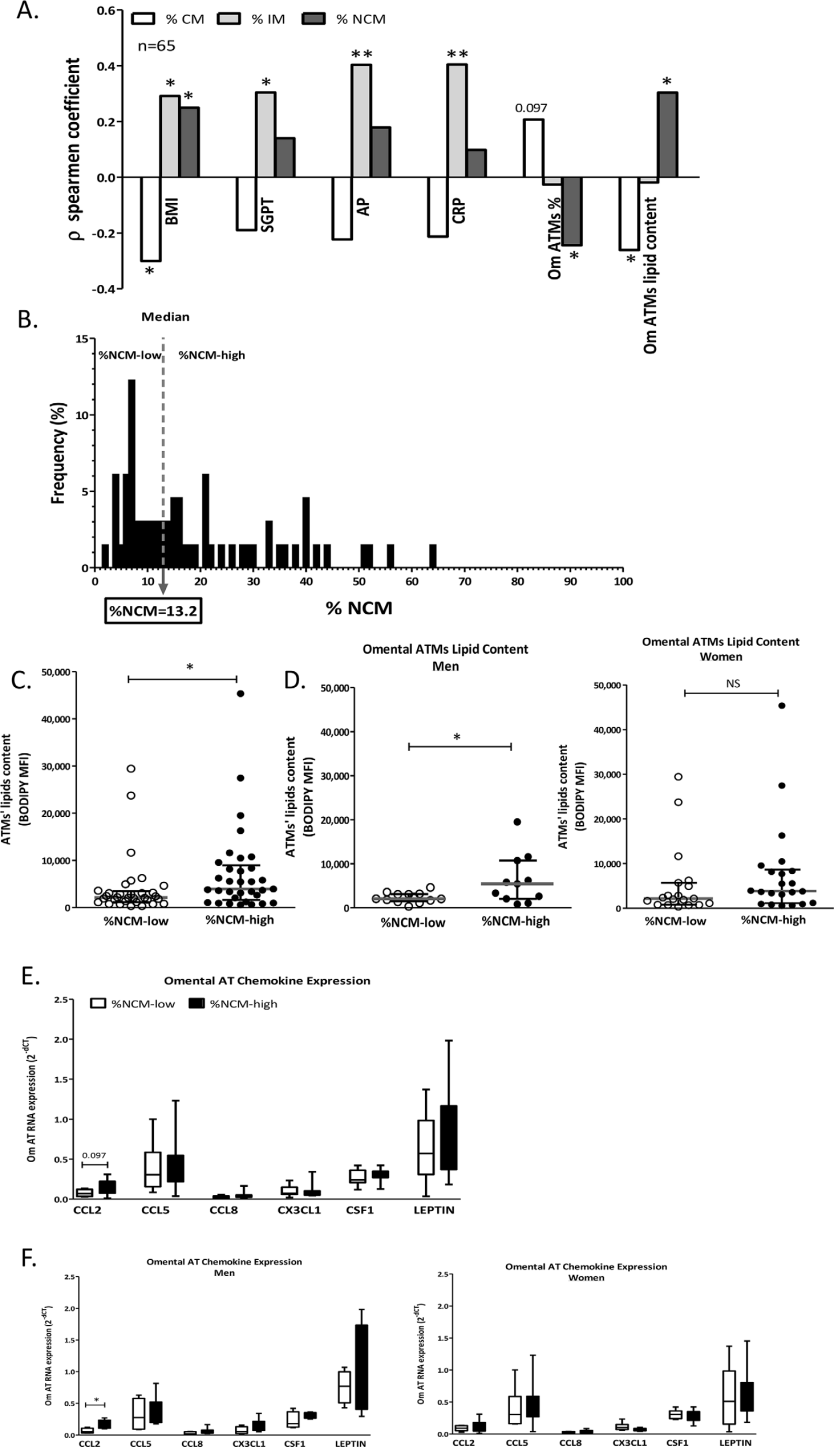
Monocyte subclasses association with clinical and adipose tissue parameters. **A)** Spearman Correlation analysis between clinical parameters and omental adipose tissue parameter and % circulating monocyte subclass (CM-classical monocytes; IM-intermediate monocytes; NCM-non-classical monocytes). **B)** Distribution of the patients' cohort to %NCM-low and–high according to the median value (13.2%). Omental ATM lipid content was compared between %NCM-low and %NCM-high **C)** in the whole cohort (n = 32 vs. n = 33, respectively), or separately **D)** in men (left graph, n = 13 vs. n = 11, respectively) and in women (right graph, n = 19 vs. n = 22, respectively). Adipose tissue chemokine expression was compared between %NCM-low and %NCM-high **E)** in the whole cohort (n = 11 vs. n = 15, respectively), or separately **F)** in men (left graph, n = 4 vs. n = 6, respectively) and in women (right graph, n = 7 vs. n = 9, respectively). Statistical comparison was obtained by the Mann-Whitney U test. *p< 0.05, **p<0.01.

Consistent with previous reports [4, 15], Spearman correlations revealed that higher BMI associated with lower percentage of CM and higher percentage of the CD16+ subpopulations—both IM and NCM (Fig 4A and S2 Data). Uniquely, only higher percentage of IM significantly correlated with higher serum liver enzymes and with CRP. Intriguingly, higher %NCM associated with lower percentage of ATM out of the entire population of adipose tissue leucocytes (CD45+ cells). This association was also evident between %NCM and ATMs in the subcutaneous fat (sub-cohort, n = 25. r = -0.405, p = 0.045). Moreover, both higher %NCM and lower %CM significantly associated with elevated ATM lipid content in omental fat (Fig 4A), but not in subcutaneous fat (p = 0.402 and p = 0.353 for %NCM and %CM, respectively). We further divided participants into those who had either low or high %NCM (below or above the median = 13.2%, respectively, Fig 4B; their respective clinical characteristics are presented in Table 1). %NCM-high patients had on average a 1.8-fold higher ATM lipid content in their omental adipose tissue (p = 0.016, Fig 4C). When dividing the cohort by sex, this effect seemed to largely be contributed by the men sub-group, mainly because of high variability in ATM lipid content among %NCM-low women (Fig 4D). Interestingly, utilizing samples from a sub-cohort of patients (n = 10 men, 16 women) adipose tissue expression of CCL2/MCP-1 (but not 5 other chemokines) was significantly higher, particularly in men, who were %NCM-high (Fig 4E and 4F).

To further challenge the apparent association between %NCM and ATM lipid content (Fig 4A) and to what degree it is independent of potential confounders, we utilized linear regression models. The association between %NCM and ATM lipid content remained statistically significant also after adjusting to participants’ gender (sex), but the model exhibited a p>0.05 when the model was further adjusted to either age or BMI (Table 2 and S2 Data). When considering only patients with BMI≥25 Kg/m^2^ (n = 51), %NCM associated with ATM lipid content independently of sex, age or BMI (Table 2). Moreover, in men the association of %NCM was even stronger (n = 24, r^2^ = 0.679, p<0.001). Expectedly, %CM exhibited significant co-linearity with %NCM, and thus negatively associated with omental ATM lipid content. Yet, unlike with %NCM, this model lost significance when adjusting for age or BMI, in both the entire cohort or in the sub-cohort of those with BMI≥25 Kg/m^2^ (S2 Table). Thus, %NCM stands out as being independently associated with increased ATM lipid content in omental fat in humans, particularly in over-weight and obese men.

**Table 2:** %NCM as a statistical predictor of omental ATM lipids content: Dependent variable ATMs' lipids content

		**Independent variable %NCM**		**Model summary**	
	**Adjustment**	**Beta**	**p-value**	R^2^	**p-value**
All (n = 65)	**-**	**0.287**	**0.021**	**0.068**	**0.021**
	**Sex**	**0.292**	**0.018**	**0.080**	**0.028**
	Age	0.283	0.023	0.056	0.063
	BMI	0.305	0.019	0.056	0.062
BMI ≥25 Kg/m^2^ (n = 51)	**-**	**0.339**	**0.014**	**0.097**	**0.014**
	**Sex**	**0.339**	**0.014**	**0.100**	**0.028**
	**Age**	**0.345**	**0.014**	**0.081**	**0.048**
	**BMI**	**0.344**	**0.014**	**0.080**	**0.048**

Abbreviations: NCM, non-classical monocytes; BMI, body mass index;

## Discussion

Unique associations between specific circulating monocyte subclasses and certain characteristics of obesity and/or its subphenotypes, including “high cardiovascular risk obesity”, have already been proposed [4, 15, 19, 20], and supported also by our cohort. Yet, the present study adds the following novel observations: i) NCM exhibit the lowest lipid accumulation capacity of the three monocyte subpopulations, both as circulating monocytes and when differentiated into macrophages *ex-vivo*. ii) Similarly, NCM migrate the least towards conditioned media from omental fat. iii) Clinically, increased %NCM associate with higher ATM lipid content in visceral fat, and in persons with BMI≥25 Kg/m^2^ this association is independent of age, sex, and importantly, is significant also beyond BMI. iv) Patients with high %NCM (i.e., above median, >13.2%) present with both higher omental ATM lipid content and higher CCL2 expression in their omental fat, particularly in men. Jointly, these findings suggest novel insights, both for mechanistic understanding of adipose tissue inflammation in obesity and potentially for health-risk–related subphenotyping of obese patients.

Our study raises the question of whether in obesity visceral lipid-laden ATMs are derived from a specific circulating monocyte subclass? The ontogeny of ATMs in obesity is still debated: although resident macrophage proliferation and polarization [21]and adipose mesenchymal stem cells differentiation have been proposed, monocytes infiltrating the tissue from the circulation are still thought to constitute a major source of ATMs. Yet, which circulating monocytes subclass dominates this process, and whether different subclasses give rise to pro-inflammatory versus remodeling macrophages, has not been established in obesity. This is despite consistent findings among studies linking increased circulating NCM in human obesity and in its associated co-morbidities [4, 15, 16]. Yet, origin of pro-inflammatory vs. remodeling macrophages may be disease-specific [22]: In injured tissues NCM (Ly6C-) may be the main contributor to the pool of remodeling macrophages [10, 23]. Conversely, when tissue inflammation “matures”, cross-differentiation of CM (Ly6C+) -derived M1 macrophages into M2 may be a predominant source of remodeling macrophages [24, 25]. Furthermore, when specifically considered adipose tissue foam cells, it is equally difficult to speculate on whether a specific monocyte subclass could be the source, since they display a mixed immunological phenotype, with joint expression of M1 and M2 markers in obese mice (CD11c and CD209, respectively)[2]. *Ex-vivo*, the three monocytes subclasses in humans present differential migratory capacity [18, 26, 27], but migration of specific monocyte subclasses towards adipose tissue-derived secreted factors has not been previously explored. We demonstrate that CM is the circulating monocyte subclass with the highest migratory capacity towards secreted products from human visceral adipose tissue. Interestingly, %NCM-high patients had lower percentage of ATM out of the total adipose tissue leucocytes (CD45+ cells) in visceral fat, and in %NCM-high men the visceral fat expressed higher mRNA levels of CCL2, a chemokine ligand for CCR2-positivie monocytes, which are likely the CM subpopulation [7, 18]. Taken together, these data may suggest that the CM subclass, and not the NCM subclass, is functionally the most likely circulating monocyte subclass to migrate towards visceral adipose tissue. Whether they then give rise to more pro-inflammatory ATM or conversely, via such cells or directly to more remodeling-type ATMs—is unknown. Nevertheless, though our work is limited to ex-vivo assays and associations, we can speculate that in humans a higher percentage of the NCM subclass in the circulation compartment could reflect greater migration of CM into visceral adipose tissue. Yet, fully appreciating the relative contribution of such a “CM depletion mechanism” (i.e., CM preferentially migrating from the circulation into the adipose tissue), awaits more understanding of the dynamics of monocyte subclass production and/or release from the bone marrow into the circulation.

The NCM presented also the lowest lipid accumulation capacity among the three subsets, either as monocytes or as adipose tissue-like monocyte-derived macrophages. This further questions the likelihood that NCM may serve as precursor cells for lipid-laden adipose tissue macrophages, and highlights the functional potential of the CM. These results can be explained by different metabolic profiles of the subsets. Indeed, in a comprehensive transcriptomics and enhancer profiling in humans, CM and NCM monocytes subclasses displayed marked differences in metabolic gene signatures: while CM expressed higher levels of genes involved in carbohydrate metabolism, priming them for glycolytic metabolism, NCM expressed higher levels of oxidative pathway components [28]. These inherent metabolic differences can influence triglycerides accumulation in the cells, and hence potentially foam cell biogenesis when these cells differentiate into tissue macrophages. Indeed, increased glucose uptake in LPS-activated macrophages was shown to be accompanied by an increase in triglycerides storage in the cells [29]. Thus, differences in monocyte/macrophage metabolism would also support CM as the more likely lipid-laden ATM precursor.

Our study may also suggest some clinically-relevant insights: adipose tissue inflammation, particularly of visceral depots, is strongly implicated as a signature of obesity phenotype(s) with high cardiometabolic risk [30, 31]. Thus, there is clinical interest to infer on visceral fat inflammatory state by clinical parameters as part of health risk-related subphenotyping of the obese patient. We propose that %NCM in circulating blood may serve as one putative predictor of visceral fat inflammatory state as reflected by ATM lipid content. Indeed, the fact that this association remained independent of BMI among overweight and obese persons supports its potential clinical usefulness in such subphenotyping, beyond the degree of obesity per-se. Nevertheless, several limitations of our study are also noteworthy: Our study is a cross-sectional analysis, and *ex-vivo* functional assessments may not truly reflect the function of monocyte subpopulations *in vivo*. Yet, the study’s strengths include the co-analysis of visceral (omental) adipose tissue samples vis-à-vis circulating blood cells in samples taken from the same patients on the same day.

In conclusion, in humans, circulating blood monocyte subclass analyses may shed light on mechanisms of visceral adipose tissue inflammation, and may assist in identifying overweight and obese persons (especially males) with particularly elevated cardiometabolic risk, beyond BMI.

## Supporting Information

S1 Fig Gating strategy for monocytes subclasses. (TIF)

S1 Table List of Taq-man primers for quantitative real time PCR (Applied Biosystems). (DOCX)

S2 Table %CM as a statistical predictor of omental ATM lipids content. (DOCX)

S1 Data Raw numbers for analyses in Figs 1 and 2. (XLSX)

S2 Data Raw numbers for analyses in Tables 1 and 2 and Fig 4. (XLSX)
