# Supplementary figures and images for: Circulating Blood Monocyte Subclasses and Lipid-Laden Adipose Tissue Macrophages in Human Obesity
Source: PLoS One. 2016 Jul 21;11(7):e0159350. doi: 10.1371/journal.pone.0159350 (PMC4956051; doi:10.1371/journal.pone.0159350)

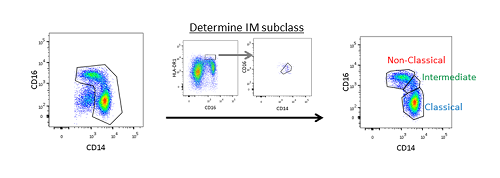

Supplement: S1 Fig — (TIF) [file pone.0159350.s001.tif]
