# Supplementary material for: Circulating Blood Monocyte Subclasses and Lipid-Laden Adipose Tissue Macrophages in Human Obesity
Source: PLoS One. 2016 Jul 21;11(7):e0159350. doi: 10.1371/journal.pone.0159350 (PMC4956051; doi:10.1371/journal.pone.0159350)
Supplement: S1 Table — (DOCX) [file pone.0159350.s002.docx]

**Supplementary table 1: List of Taq-man primers for quantitative real time PCR (Applied Biosystems)**

| **Reference** | **Gene** |
| --- | --- |
| Hs00234140_m1 | CCL2 |
| Hs00982282_m1 | CCL5 |
| Hs04187715_m1 | CCL8 |
| Hs00174877_m1 | LEP |
| Hs00171086_m1 | CX3CL1 |
| Hs00174164_m1 | CSF1 |
| Hs99999904_m1 | PPIA |
| Hs00943178_g1 | PGK1 |
