# Supplementary material for: Circulating Blood Monocyte Subclasses and Lipid-Laden Adipose Tissue Macrophages in Human Obesity
Source: PLoS One. 2016 Jul 21;11(7):e0159350. doi: 10.1371/journal.pone.0159350 (PMC4956051; doi:10.1371/journal.pone.0159350)
Supplement: S2 Table — (DOCX) [file pone.0159350.s003.docx]

***%CM as a statistical predictor of omental ATM lipids content:***

*Dependent variable ATMs' lipids content*

| **Model summary** | | **Independent variable %CM** | |  |  |
| --- | --- | --- | --- | --- | --- |
| **p-value** | **R^2^** | **p-value** | **Beta** | **Adjustment** |  |
| 0.025 | 0.063 | 0.025 | -0.278 | - | All (n=65) |
| 0.037 | 0.072 | 0.024 | -0.278 | Sex |  |
| 0.077 | 0.050 | 0.029 | -0.273 | Age |  |
| 0.072 | 0.052 | 0.023 | -0.298 | BMI |  |
| 0.016 | 0.092 | 0.016 | -0.332 | - | BMI ≥25 Kg/m_2_ (n=51) |
| 0.036 | 0.092 | 0.018 | -0.327 | Sex |  |
| 0.052 | 0.078 | 0.016 | -0.342 | Age |  |
| 0.054 | 0.076 | 0.016 | -0.339 | BMI |  |
|  |  |  |  |  |  |

*Abbreviations: CM, classical monocytes; BMI, body mass index;*
